# Molecular Crosstalking among Noncoding RNAs: A New Network Layer of Genome Regulation in Cancer

**DOI:** 10.1155/2017/4723193

**Published:** 2017-09-24

**Authors:** Marco Ragusa, Cristina Barbagallo, Duilia Brex, Angela Caponnetto, Matilde Cirnigliaro, Rosalia Battaglia, Davide Barbagallo, Cinzia Di Pietro, Michele Purrello

**Affiliations:** ^1^BioMolecular, Genome and Complex Systems BioMedicine Unit (BMGS Unit), Section of Biology and Genetics G Sichel, Department of BioMedical Sciences and Biotechnology, University of Catania, Catania, Italy; ^2^IRCCS Associazione Oasi Maria S.S., Institute for Research on Mental Retardation and Brain Aging, Troina, Enna, Italy

## Abstract

Over the past few years, noncoding RNAs (ncRNAs) have been extensively studied because of the significant biological roles that they play in regulation of cellular mechanisms. ncRNAs are associated to higher eukaryotes complexity; accordingly, their dysfunction results in pathological phenotypes, including cancer. To date, most research efforts have been mainly focused on how ncRNAs could modulate the expression of protein-coding genes in pathological phenotypes. However, recent evidence has shown the existence of an unexpected interplay among ncRNAs that strongly influences cancer development and progression. ncRNAs can interact with and regulate each other through various molecular mechanisms generating a complex network including different species of RNAs (e.g., mRNAs, miRNAs, lncRNAs, and circRNAs). Such a hidden network of RNA-RNA competitive interactions pervades and modulates the physiological functioning of canonical protein-coding pathways involved in proliferation, differentiation, and metastasis in cancer. Moreover, the pivotal role of ncRNAs as keystones of network structural integrity makes them very attractive and promising targets for innovative RNA-based therapeutics. In this review we will discuss: (1) the current knowledge on complex crosstalk among ncRNAs, with a special focus on cancer; and (2) the main issues and criticisms concerning ncRNAs targeting in therapeutics.

## 1. Introduction

When the Human Genome Project (HGP) began in the late 1990s, researchers hypothesized that our genome comprised about 100,000 protein-coding genes [1]. Over the years, this estimate has been continuously downsized. In 2001, the International Human Genome Sequencing Consortium (IHGSC) published the initial sequence of the human genome and proposed that the number of protein-coding genes was about 30,000 [2]. At the same time, Celera Genomics (a competitor group of IHGSC) estimated this number at 26,000 [3]. In 2004, when the final draft of the human genome was published, this number was further reduced to 24,500 [4], but in 2007 an additional analysis established that it was around 20,500 [5]. More recently, new studies updated the number of human protein-coding genes to 19,000 [6]. This estimate is particularly surprising, because it would suggest that less than 2% of the whole human genome encodes for proteins; accordingly, the keystone of *Homo sapiens* complexity could lie in the 98% of our DNA (the genome *dark matter*), which does not encode proteins but would be endowed with critical regulatory functions. In the last decade, two important scientific initiatives supported by the US National Institutes of Health (i.e., the projects ENCODE and Roadmap Epigenomics) reported seminal data on hundreds of thousands of functional regions in the human genome, whose function is to supervise gene expression [7, 8]. These data suggested that much more space in our genome is committed to regulatory than to structural functions. Moreover, these studies proposed that about 80% of the human genome is dynamically and pervasively transcribed, mostly as non-protein-coding RNAs (ncRNAs). The biological relevance of the noncoding transcriptome has become increasingly undeniable over the last few years. Studies of comparative genomics showed that the relative proportion of genome space, occupied by the proteome-encoding genome as opposed to the regulatory (non-protein-encoding) genome is very variable among evolutionarily distant species; for instance, the protein-coding genome represents almost the entire genome of the unicellular yeast *Saccharomyces cerevisiae*, whereas it constitutes only 2% of mammalian genomes [9]. Moreover and intriguingly, the noncoding transcriptome is frequently altered in major diseases, including cancer [10–12]. These observations strongly suggest that ncRNAs are closely related to the complexity of higher eukaryotes and that their dysfunction may result in pathological phenotypes. RNA is a structurally versatile molecule, able to perform several molecular functions. By simple base pairing with other nucleic acids, RNA can recognize and bind both DNA and RNA targets in a very specific manner and regulate their transcription, processing, editing, translation, or degradation. An intriguing field for future explorations is the tridimensional folding of RNA molecules, which confers them allosteric properties: this increases the range of potential molecular interactors (including proteins); additionally, dynamic conformational changes can be triggered by ligand binding. Moreover and different from proteins, RNA can be rapidly transcribed and degraded making it a very dynamic molecule that can be quite rapidly synthesized without additional time and energetic costs of translation [13]. For all these reasons, over the past few years, ncRNAs have been extensively studied because of the significant biological roles that they play in regulation of cellular mechanisms. Noncoding RNA genes can generally be divided into two major categories by their transcript sizes: (1) long noncoding RNAs (lncRNAs) are longer than 200 nucleotides; and (2) small noncoding RNAs have a length equal to or lower than 200 nucleotides [i.e., microRNAs (miRNAs), small interfering RNAs (siRNAs), small nuclear RNAs (snRNAs or U-RNAs), small nucleolar RNAs (snoRNAs), PIWI-interacting RNAs (piRNAs), and tRNAs] [14]. To date, most research efforts have been focused on how ncRNAs (in particular, miRNAs) modulate the expression of protein-coding genes and their roles in human pathophysiology. However, recent evidence has shown the existence of unexpected interplay among ncRNAs, which influences cell physiology and diseases. In addition to the canonical multilayered control of expression of protein-coding genes (briefly described below), ncRNAs can interact with and regulate each other through various molecular mechanisms generating a complex network including different species of RNAs. In such a regulatory network, ncRNAs also compete among each other for binding to mRNAs, thus acting as competing endogenous RNAs (ceRNAs). In this review, we will summarize the current knowledge on the complex crosstalk among ncRNAs (including miRNAs, lncRNAs, and circRNAs) and how they could reciprocally interact to regulate cancer progression and dissemination.

### 1.1. miRNAs

miRNAs are 18–25 nucleotides long, evolutionarily conserved, single-stranded RNAs, which negatively modulate the expression of their target mRNAs (more than 60% of protein-coding genes) by binding to the 3′-UTR of specific mRNA targets, leading either to their translational repression, cleavage, or decay [15–17]. This binding occurs through a specific miRNA region (named *seed region*), which is a contiguous string of at least 6 nucleotides beginning at position two of the 5′ of the molecule [18]. The block of translation is due to the inhibition of mRNA 5′-cap recognition and interference on the interaction between the mRNA and the 60S ribosomal subunit, while mRNA degradation is promoted by mechanisms of decapping and deadenylation [19]. These molecular mechanisms are mediated by an RNA-induced silencing complex (RISC) that includes proteins belonging to the Argonaute (AGO) family; specifically, RISC endonuclease activity depends exclusively on AGO2 protein [20]. A single miRNA can control the expression of several mRNAs, and a single mRNA may be targeted by more than one miRNA, thus creating a complex interplay of cooperative regulation [21]. To date, more than 2500 mature miRNAs have been included in the *miRbase* database [22].

Extensive studies have shown that miRNAs control pivotal cellular processes, (e.g., cell proliferation, differentiation, migration, cell death, and angiogenesis), thus contributing to the pathogenesis of diseases such as cancer. Indeed, several miRNAs have been identified as potential oncogenes or tumor suppressors in cancer development and progression [23]. In the last two decades, their mutations and altered expression were reported to be causally related to the neoplastic features of the cells, thus providing new perspectives for the understanding of the complex regulatory networks that rule tumor biology [24]. miRNA dysfunctions exert a pleiotropic effect on the expression of their mRNA targets impairing the functioning of biological networks. It has been convincingly demonstrated that different cancer histotypes display specific miRNA expression patterns: this phenomenon would be helpful to improve diagnosis of poorly differentiated tumors and predict prognosis in cancer [25, 26]. Moreover, multiple experimental evidence has shown that miRNAs can be also secreted by cancer cells into bodily fluids, sending oncogenic signals through circulation, which could advantageously mold the extracellular tumor environment [27]. These discoveries gave a new intriguing diagnostic and prognostic role to circulating miRNAs, paving the way for their potential use as noninvasive molecular RNA markers in clinical management of cancer patients [28–30].

### 1.2. lncRNAs

lncRNAs are the most heterogeneous class of non-protein-coding RNAs with lengths ranging from 200 nt to 100,000 nt. They include transcripts that may be classified as (a) intergenic lncRNAs, (b) intronic lncRNAs, (c) sense or antisense transcripts, (d) pseudogenes, and (e) retrotransposons [14]. Currently, LNCipedia 4.0 records more than 118,000 human lncRNAs, which are usually expressed in a developmental and tissue-specific manner [31]. lncRNAs regulate gene expression at different levels, including chromatin modification, alternative splicing, and protein localization and activity [32]. Such a wide range of mechanisms is due to their ability to bind to DNA, RNAs, and proteins. lncRNAs, thanks to their binding to promoter DNA, can prevent the access of transcription factors to their own promoter binding sites and impede the transcription of specific genes (e.g., DHFR) [33]. Some lncRNAs (e.g., HOTAIR) are associated with chromatin-modifying complexes (e.g., polycomb repressive complex 2) to regulate epigenetic silencing of target genes [34]. Much evidence has also shown that lncRNAs may work as molecular scaffolds to connect two or more proteins in functional complexes or can serve to localize protein complexes to appropriate cellular compartments [35]. Antisense lncRNAs can target, by direct sequence complementarity, their antisense mRNAs and, accordingly, modulate alternative splicing processes or protect 3′-UTR from miRNA binding, increasing the stability of mRNAs (e.g., ZEB2-AS1, BACE1-AS) [36, 37]. Several recent studies have shown that lncRNAs are critically involved in a wide range of biological processes, such as cell cycle regulation, pluripotency, differentiation, and cell death [38–41]. Dysregulation of lncRNA activity has been frequently reported in association to diseases, including several types of cancer. Specifically, upregulated lncRNAs in cancer seem to possess tumor-promoting abilities, whilst downregulated lncRNAs exhibit tumor-suppressive roles [42–47]. Although several lncRNAs have been reported to be dysregulated in neoplastic phenotypes, their mechanistic role in cancer biology has not been satisfactorily explained for most of them. However, scientific evidence strongly suggests a promising role for lncRNAs as cancer-related biomarkers and potential targets for innovative therapeutic approaches.

### 1.3. circRNAs

Circular RNAs (circRNAs) represent a recently discovered class of noncoding RNAs, composed of single-stranded, covalently closed, exonuclease-resistant circular transcripts [48]. Although the existence of circular RNAs has been known since the 70s [49], for a long time such molecules were considered only by-products of pre-mRNA processing and therefore interpreted as artifacts of aberrant RNA splicing [50]. However, recent advances in RNA sequencing technologies have revealed a ubiquitous, and in some cases abundant, expression of endogenous circRNAs in mammalian genomes [51, 52]. circRNAs are a circularized isoform of linear protein-coding genes generated through backsplicing, a molecular process that is different from the canonical splicing of linear RNAs. Circular RNA biogenesis can occur both from exons (exonic circRNAs or ecircRNAs), through different mechanisms of backsplicing and introns (intronic circRNAs or ciRNAs), when lariat introns escape typical debranching processes [53]. Currently, about 35,000 circRNAs are reported in the *circBase* database [54], but molecular functions and biological processes, in which they are involved, remain elusive for most of them. Recent emerging evidence convincingly suggests that circRNAs may play an important role in RNA-RNA interactions. In some instances, circRNAs exhibit multiple binding sites for the same miRNA and represent a potential *molecular sponge* for sequestering the most abundant miRNAs [55]. In other words, circRNAs may negatively regulate the function of miRNAs, and, thus, protect miRNA targets, by acting as competing endogenous RNAs. As some papers would suggest that ceRNA role of circRNAs could not be their main function in cell biology, other molecular functions have been proposed for circRNAs (a) to bind and sequester RNA binding proteins (RBPs) [56–58] and (b) to be translated into proteins when recognized by ribosomes in the presence of internal ribosome entry sites (IRESs) [59, 60]. As circRNAs are potentially able to control different layers of gene expression, it is not surprising that their dysregulation is associated with human pathologies, including cancer [61–63]. Most reports that connect circRNAs and tumors mainly concern comparative gene expression profiling studies between tumor and normal samples. These investigations have shown that circRNAs are frequently downregulated in several types of cancer (e.g., colorectal cancer, ovarian cancer, and gastric cancer) [64–66]. Just few of these studies attempted to functionally explain how abnormal expression of circRNAs could impair physiological cell homeostasis and thus promote cancer phenotypes [67–69].

## 2. Noncoding RNAs: Different Ways to Interplay among Each Other

Interplay between ncRNAs obviously occurs because of sequence complementarity; for instance, ncRNAs may share miRNA response elements (MREs) with mRNAs and thus be targeted in the same manner [70]. The effects of miRNAs binding to other ncRNAs (i.e., lncRNAs and circRNAs) could be twofold: on the one hand, miRNAs could be sequestered and prevented from acting on the protein-coding mRNAs; on the other hand, miRNA binding to lncRNAs and circRNAs could promote their decay, similarly to mRNAs. In the next paragraphs, we will discuss the different mechanisms of ncRNA interaction and their influence on cancer biology.

### 2.1. miRNAs Induce Degradation of lncRNAs

Several papers have reported that miRNAs can bind lncRNAs and promote their degradation contributing to cancer processes (Table 1). lncRNAs are structurally similar to mRNAs; indeed, they have 5′-caps and 3′-poly(A) tails [71]; accordingly, the proteins involved in the regulation of decapping, deadenylation, and degradation of mRNAs may also control the turnover of lncRNAs by binding of specific miRNAs.

UCA1 (urothelial cancer associated 1), an lncRNA upregulated in several tumors (i.e., bladder cancer, tongue squamous cell carcinoma, breast cancer, and ovarian cancer) [72–75], possesses two predicted binding sites for miR-1, a well-known tumor suppressor miRNA. The binding of miR-1 to UCA1 has been confirmed by luciferase reporter assay in bladder cancer and, accordingly, *in vitro* upregulation of miR-1 induced UCA1 downregulation and caused a decreased cell growth and migration and also an augmented apoptosis. Such functional effects were reverted after UCA1 overexpression and silencing of AGO2, suggesting that miR-1 was able to downregulate UCA1 expression in an AGO2-mediated manner [76].

MALAT1 (metastasis-associated lung adenocarcinoma transcript 1) is one of the most studied and abundant lncRNAs: its expression was initially associated with metastasis in non-small-cell lung carcinoma (NSCLC) [77], but then its deregulation has been reported in several other neoplastic diseases [78–80]. The 3′ end of MALAT1 is cleaved by RNase P and RNase Z, producing a tRNA-like ncRNA, called mascRNA (MALAT1-associated small cytoplasmic RNA), which will be exported into the cytoplasm [81], while most of the MALAT1 molecules are localized to nuclear speckles where they regulate alternative splicing of specific pre-mRNAs [82]. Moreover, MALAT1 may bind CBX4 (chromobox 4), a component of polycomb repressive complex 1 (PRC1), and modulate its localization in interchromatin granules, leading to activation or inhibition of gene expression [83]. Through these molecular mechanisms, MALAT1 controls the expression of several genes related to cell cycle and metastatic processes, thus influencing cell proliferation, migration, and invasion. Recent publications reported that MALAT1 is a target of a number of tumor suppressor miRNAs, which could induce its degradation and suppress its oncogenic effects. Leucci et al. reported miRNA-mediated regulation of MALAT1 in the nucleus of Hodgkin lymphoma and glioblastoma cell lines through direct binding of miR-9 to two different MREs in an AGO2-dependent manner [84]. There is evidence of a posttranscriptional regulation of MALAT1 by miR-101 and miR-217 in esophageal squamous cell carcinoma (ESCC) cells [85]. MiR-101 and miR-217 are functionally involved in several cancers as tumor suppressors and exhibited a significant negative correlation with MALAT1 in ESCC tissue samples and adjacent normal tissues. Enforced expression of miR-101 and miR-217 significantly repressed MALAT1 expression, leading to inhibition of cell growth, invasion, and metastasis in ESCC cells [85]. In bladder cancer, MALAT1 is inversely expressed with miR-125b. This miRNA was partially complementary with MALAT1 and bound it in *in vitro* models. MiR-125b was downregulated in bladder cancer, and its overexpression decreased the expression of MALAT1, causing an inhibition of bladder cancer cell proliferation, motility, and activation of apoptosis [86].

Additionally, miR-125b was also identified as a posttranscriptional regulator of HOTTIP (HOXA distal transcript antisense RNA) in hepatocellular carcinoma (HCC) [87]. HOTTIP is one of the most upregulated lncRNAs in HCC, also in early stages of HCC onset, and maps in antisense position to the distal end of the HOXA gene cluster. HOTTIP promotes tumor growth and metastasis *in vitro* and *in vivo* through regulation of the expression of its neighboring HOXA genes (e.g., HOXA10, HOXA11, and HOXA13). MiR-125b has been reported to be frequently downregulated in HCC, and a negative correlation of expression between miR-125b and HOTTIP existed in such cancer. The interaction between miR-125b and HOTTIP was validated by luciferase reporter assay; this was confirmed by ectopic expression of miR-125b that induced downmodulation of HOTTIP [87].

HOTAIR (HOX antisense intergenic RNA) is one of the most intensively studied lncRNAs, as it is frequently associated with different neoplasias. HOTAIR exerts its oncogenic functions by working as a scaffold to assemble polycomb repressive complex 2 (PRC2) on the HOXD gene cluster and inducing the transcriptional silencing of multiple metastasis suppressor genes (e.g., the protocadherin gene family) [34, 88]. HOTAIR is posttranscriptionally destabilized by several tumor suppressor miRNAs in different cancers. Chiyomaru et al. reported a functional binding between miR-34a and HOTAIR in prostate cancer cell lines treated with genistein, an isoflavone with antitumor activity: miR-34a directly bound to two MREs within HOTAIR RNA and lowered its levels [89]. Yoon et al. reported that human antigen R (HuR), let-7b, let-7i, and AGO2 cooperatively bind HOTAIR and promote HOTAIR decay, thus inhibiting the processes of ubiquitination and proteolysis of Ataxin-1 and Snurportin-1, promoted by HOTAIR [90]. Interestingly, HuR and let-7b/AGO2 complex also decreased the stability of lincRNA-p21, an oncogenic lncRNA that reduced translation of beta-catenin and JUNB (JunB proto-oncogene, subunit of transcription factor AP-1) mRNAs in human cervical carcinoma HeLa cells [91]; even if in other experiments HuR was not able to transfer let-7b to AGO2 [92]. In another paper by Chiyomaru et al., it was reported that HOTAIR expression is negatively correlated to that of miR-141 in renal carcinoma cells (RCC) [93]. MiR-141 belongs to the miRNA-200 family, which has been reported to inhibit epithelial-mesenchymal transition (EMT) by ZEB1 (zinc finger E-box-binding homeobox 1) repression and E-cadherin upregulation [94]. MiR-141 was able to target and cleave HOTAIR in an AGO2-dependent manner, and such molecular action downregulated the expression of ZEB2 (zinc finger E-box-binding homeobox 2) induced by HOTAIR [93].

Expression of miR-141 was also found to be negatively correlated to that of lncRNA H19 (H19, imprinted maternally expressed transcript) in gastric cancer [95]. H19, an oncofetal lncRNA, is highly expressed during embryogenesis [96] and is upregulated in several cancers, including gastric cancer [97]. H19 acts as the primary miRNA precursor of miR-675, which in turn targets and represses RB1 (RB transcriptional corepressor 1) mRNA [98]. Overexpression of H19 enhances tumor cell growth and induces EMT; additionally, H19 modulates miRNA processing through its interaction with proteins involved in this molecular process (i.e., Drosha, Dicer). MiR-141 was shown to bind H19 in gastric cancer, and suppress H19 expression and its tumor-promoting functions [95].

MiR-21 is the most commonly upregulated miRNA in cancer: its genetic locus is often amplified in solid tumors, and its expression is promoted by a variety of cancer-related *stimuli* [99]. MiR-21 enhances cell proliferation, migration, and invasion by targeting several tumor suppressor genes, such as CCL20, CDC25A, PDCD4, and PTEN [100–103]. Recent findings showed that some lncRNAs could be added to the *repertoire* of miR-21 targets. Zhang et al. reported that expression of miR-21 and lncRNA GAS5 (growth arrest-specific 5) is negatively correlated in breast cancer and that miR-21 binds a miR-21-binding site in exon 4 of GAS5, thus inducing AGO2-mediated suppression of GAS5 [104]. GAS5 is an lncRNA with tumor-suppressive properties: its overexpression sensitizes cancer cells to UV or doxorubicin and decreases tumor proliferation and cell invasion. Interestingly, GAS5 also negatively regulated miR-21 at the posttranscriptional level through the RISC complex, suggesting the existence of a reciprocal negative feedback loop between GAS5 and miR-21 [104]. In two different studies on renal cell carcinoma and glioblastoma, it has been shown that miR-21 targeted and suppressed the expression of the tumor suppressor lncRNA CASC2 (cancer susceptibility candidate 2) in an AGO2-dependent manner [105, 106]. Indeed, the overexpression of miR-21 abrogated the inhibition of proliferation, migration, and the induction of apoptosis promoted by CASC2. Notably, when CASC2 was upregulated, miR-21 expression decreased: this suggests reciprocal repression between miR-21 and CASC2 [106].

The first experimental evidence that lncRNAs may be targeted by miRNAs was reported for the antisense transcript of the cerebellar degeneration-related protein 1 (CDR1, also known as CiRS-7 or CDR1AS), which is a circular RNA produced by a backsplice event [107]. MiR-671, a nuclear-enriched miRNA, induced cleavage of CDR1AS in an AGO2-dependent manner. Repression of miR-671 promoted the upregulation of both CDR1AS and CDR1, suggesting that CDR1AS was able to stabilize the sense transcript CDR1. Currently, this represents the only report on circRNA targeted and degraded by a miRNA. The interaction between miR-671 and CDR1AS could affect the biopathological molecular asset of glioblastoma multiforme (GBM), the most prevalent and aggressive cancer originating in the central nervous system, mainly in the brain. Indeed, Barbagallo et al. demonstrated that miR-671-5p is significantly upregulated in GBM. Enforced expression of miR-671-5p increased migration and decreased proliferation rates of GBM cell lines, suggesting its potential role as a novel oncomiRNA in GBM [108]. Expression of miR-671 was inversely correlated to that of CDR1AS and CDR1 in GBM biopsies and the expression of CDR1AS and CDR1 decreased when the miR-671 mimic was used, suggesting that the interaction of these molecules could be functionally altered in a GBM model [108].

### 2.2. lncRNAs as Decoys of miRNAs

The most explored mechanism of functional interactions between lncRNAs and miRNAs is based on sharing the same miRNA target sequence in both lncRNAs and mRNAs. In this way, lncRNAs are able to sequester miRNAs away from mRNAs, functioning as “miRNA sponges” or “miRNA decoys.” Through such a competitive endogenous mechanism of interaction, lncRNAs decrease the quantity of available miRNAs and increase, accordingly, translations of their mRNA targets. lncRNAs, working as *competitive endogenous* RNAs, have been extensively described in molecular circuits involved in tumors (Table 2).

EWSAT1 (Ewing sarcoma-associated transcript 1) is an lncRNA with oncogenic functions in Ewing's sarcoma and nasopharyngeal carcinoma (NPC). EWSAT1 has two MREs for the miR-326/330-5p cluster and promoted the development and progression of tumors functioning as a ceRNA for these miRNAs, which in turn induced the expression of Cyclin D1, target of miRNAs from the miR-326/330-5p cluster [109].

Xia et al. showed that both lncRNA FER1L4 (FER-1-like family member 4, pseudogene) and PTEN (phosphatase and tensin homolog) mRNA had binding sites for oncomiR miR-106a-5p and were downregulated in gastric cancer [110]. As FER1L4 behaved as a ceRNA for miR-106a-5p, FER1L4 downregulation released miR-106a-5p that targeted PTEN mRNA, reducing its expression. Dysregulation of FER1L4-miR-106a-5p-PTEN axis increased cell proliferation by promoting the G0/G1 to S phase transition [110].

FTH1P3 (ferritin heavy chain 1 pseudogene 3) has been shown to function as a molecular sponge for miR-224-5p in oral squamous cell carcinoma (OSCC) [111]. Overexpression of FTH1P3 promoted proliferation and colony formation in OSCC cells and the upregulation of FZD5 (frizzled class receptor 5), target of miR-224-5p and an oncogene involved in activation of Wnt/*β*-catenin signaling.

It has been demonstrated that lncRNA GAS5 acts as a tumor suppressor in NSCLC by targeting and suppressing miR-135b [112]. GAS5 is downregulated in NSCLC and its expression is inversely correlated to that of miR-135b. After exposure to irradiation, expression of GAS5 and miR-135b was altered, as GAS5 was overexpressed whereas miR-135b was downregulated. Ectopic overexpression of GAS5 led to miR-135b downregulation, repression of cell proliferation, invasion, and improved radiosensitivity [112].

High expression of lncRNA H19 in breast cancer stem cells (BCSCs) is functionally critical for stemness maintenance [113]. In these cells, H19 functions as a molecular sponge for let-7a/b, leading to upregulation of pluripotency factor LIN28, a let-7 target that is highly abundant in BCSCs. Intriguingly, H19 is reciprocally repressed by its targets let-7a/b, but this negative feedback loop can be interfered with by LIN28 because of its ability to inhibit let-7a/b expression [113]. Let-7b expression is also buffered by lncRNA HOST2 (human ovarian cancer-specific transcript 2) in ovarian cancer cells. By binding to let-7b, HOST2 negatively regulates its availability and induces the expression of its oncogenic targets that enhance cell growth and motility in ovarian cancer [114].

Let-7 decoy by lncRNAs was also reported by Deng et al. Upregulation of lncRNA CCAT1 (colon cancer associated transcript 1) in HCC tissues was associated with increased cell proliferation and migration [115]; these oncogenic activities were mediated by its molecular sponge function for let-7: inhibition of let-7 caused upregulated expression of let-7 targets: HMGA2 (high mobility group AT-hook 2) and MYC (MYC proto-oncogene, bHLH transcription factor). Interestingly, other studies reported that MYC, by binding to CCAT1 promoter, induces CCAT1 transcription in colon cancer and gastric carcinoma [116, 117], suggesting the existence of a positive feedback loop between CCAT1 and MYC mediated by let-7 decoy.

Recent works reported the inhibitory effect of HOTAIR on miRNAs functions in different neoplasias. Su et al. found that HOTAIR was highly expressed in HCC tissues and promoted HCC cell proliferation and progression of tumor xenografts [118]. These oncogenic effects were partially due to HOTAIR ability of repressing miR-1 expression. Moreover, also miR-1 was able to negatively regulate HOTAIR expression, thus generating a reciprocal repression feedback loop between these two ncRNAs [118]. Other experimental evidence showed that HOTAIR was capable of binding and downregulating miR-152 in gastric cancer [119]. HOTAIR overexpression in gastric cancer tissues led to decreased expression of miR-152 and to upregulation of its target, HLA-G (human leukocyte antigen G), which in turn facilitated tumor escape mechanisms [119]. Downregulation of miR-152 in gastric cancer could be also caused by PVT1 (plasmacytoma variant translocation 1), an oncogenic lncRNA that acts as a precursor of six miRNAs (i.e., miR-1204, miR-1205, miR-1206, miR-1207-5p, miR-1207-3p, and miR-1208) [120]. Indeed, PVT1 had three MREs for miR-152 and suppressed its expression inducing the upregulation of miR-152 targets (i.e., CD151, FGF2) [121]. Upregulation of PVT1 in gastric cancer was also associated with inhibition of miR-186 function. Indeed, PVT1 bound miR-186 and induced upregulation of HIF-1*α* (Hypoxia-inducible factor 1-alpha subunit), a target of miR-186 which was related to poor prognosis and invasiveness in gastric cancer [122].

Wang et al. studied in liver cancer the molecular sponge action of lncRNA HULC (highly upregulated in liver cancer). HULC was able to downregulate several miRNAs, including miR-372. Repression of miR-372 enhanced the translation of its target gene, PRKACB (protein kinase cAMP-activated catalytic subunit beta), which in turn promoted phosphorylation of protein CREB1 (cAMP responsive element-binding protein-1) and affected deacetylation and methylation of histones [123]. This process resulted in alterations of chromatin organization and increased expression of HULC, thus showing that HULC was involved in an autoregulatory loop that mantained its abundant expression in liver cancer [123].

Jin et al. reported an association between MALAT1 upregulation and tumor growth and metastasis in triple-negative breast cancer (TNBC) tissues [124]. These tumorigenic properties of MALAT1 were mediated by its ability to decoy miR-1 and, consequently, increase the expression of miR-1 target, SNAI2 (snail family transcriptional repressor 2), also named Slug, an oncogene involved in regulation of cancer cell invasion. Moreover, overexpression of miR-1 was able to reduce MALAT1 expression, demonstrating a reciprocal negative loop between lncRNA and miRNA [124]. The miRNA sponge function of MALAT1 was also reported for cervical cancer [125]. Indeed, MALAT1 levels were found to be more abundant in radio-resistant than in radio-sensitive cancers. Moreover, expression of MALAT1 and of its potential binding partner, miR-145, reverted in response to irradiation. The authors demonstrated that there was a reciprocal repression between MALAT1 and miR-145, which regulated the molecular mechanisms of radio-resistance of cervical cancer [125].

Notably, tumor suppressor miR-145 was frequently reported to be buffered by lncRNAs in cancer models. MiR-145 negatively regulated cell invasion in TNBC, and its downregulation was related to overexpression of lincRNA-RoR (long intergenic ncRNA Regulator of Reprogramming), which acted as competitive endogenous RNA for miR-145 [126]. LincRNA-RoR-mediated downregulation of miR-145 led to upregulation of ARF6 (ADP-ribosylation factor 6), which is strongly involved in metastatic processes; indeed, ARF6 affected E-cadherin localization and impaired cell-cell adhesion, promoting cell invasion in TNBCs [126]. Zhou et al. reported a further effective interaction between lincRNA-RoR and miR-145 in endometrial cancer. Linc-RoR functioned as a miR-145 sponge by repressing the miRNA-mediated degradation of core stem cell transcription factors (i.e., Nanog, Oct4, and Sox2), thereby maintaining the pluripotency of endometrial cancer stem cells [127].

Decoying of miR-145 was performed also by TUG1 (taurine upregulated 1), which is a well-known oncogenic lncRNA, frequently upregulated in cancer and functionally related to several aggressive features of tumors. In bladder cancer, TUG1 decreased the expression of miR-145 and caused upregulation of ZEB2, miR-145 target, promoting EMT, and increasing the metastatic proneness of bladder cancer cells [128]. The ceRNA role of TUG1 was also proved in other tumors. Overexpression of TUG1 was involved in glioblastoma angiogenesis by modulation of endothelial cell proliferation, migration, and tube formation. These cellular processes were mediated by TUG1 interaction with miR-299, which was downregulated in glioblastoma. In fact, knockdown of TUG1-induced upregulation of miR-299 and concomitant decrease of VEGFA (vascular endothelial growth factor A), target of miR-299. These molecular events resulted in a reduced tumor microvessel density in xenograft glioblastoma models [129]. Ma et al. showed that upregulation of TUG1 in gallbladder carcinoma (GBC) was related to GBC cell proliferation and metastasis, and such oncogenic activities were, at least partly, due to the sponge activity of TUG1 that bound miR-300 and negatively regulated its expression [130]. In osteosarcoma, TUG1 acted as a ceRNA by sponging miR-9-5p, inducing the upregulation of transcription factor POU2F1 (POU class 2 homeobox 1) [131]. POU2F1 is frequently upregulated in osteosarcoma and is involved in cell proliferation, differentiation and immune and inflammatory processes. Because POU2F1 is a target of miR-9-5p, silencing of TUG1-inhibited cell proliferation and colony formation, while inducing G0/G1 cell cycle arrest and apoptosis. These cellular processes were mediated by upregulation of miR-9-5p and repression of POU2F1 expression [131].

The tumor suppressor TUSC7 (tumor suppressor candidate 7; also named LOC285194) is an lncRNA transcriptionally induced by TP53 (tumor protein 53); it was initially discovered as depleted in osteosarcoma, inducing abnormal proliferation of osteoblasts, and associated with poor survival of osteosarcoma patients. Competitive endogenous binding between TUSC7 and onco-miRNAs has been frequently reported as associated with cancer-related processes. Wang et al. studied the biopathological meaning of strong downregulation of TUSC7 in HCC [132]. They found that ectopic expression of TUSC7 inhibited cell metastasis, invasion, and EMT, by functioning as a competitive sponge for miR-10a. Moreover, this miRNA was able to promote the EMT process in HCC through directly binding and repressing EPHA4 (EPH tyrosine kinase receptor A4) [132]. Moreover, exon 4 of TUSC7 harbors two binding sites for miR-211 [133]. In colon cancer, miR-211 enhanced cell growth, but this effect was reverted by enforced expression of TUSC7, which buffered the activity of miR-211 [133]. The tumor suppressor role of TUSC7 was also demonstrated in gastric cancer. TUSC7, downregulated in gastric cancer, was an independent prognostic marker of disease-free survival in patients, and its ectopic expression suppressed cancer cell growth both in *in vitro* and *in vivo* models, in part by negatively regulating the expression of miR-23 [134].

Unquestionably, one of the most iconic lncRNA acting as miRNA sponge is UCA1, which was reported to bind and repress several miRNAs in multiple tumors. UCA1 binding to miR-143 was proved in breast cancer, where UCA1 was able to modulate cell growth and apoptosis by downregulating miR-143: this in turn led to upregulation of BCL2 (BCL2, apoptosis regulator) and ERBB3 (erb-b2 receptor tyrosine kinase 3) [135]. The role of UCA1 in bladder cancer was associated with ROS (reactive oxygen species) metabolism [136]. Silencing of UCA1 decreased ROS production and promoted mitochondrial glutaminolysis in bladder cancer cells. In these cells, UCA1 acted as a ceRNA by sponging and downregulating miR-16. This induced the upregulation of GLS2 (Glutaminase 2), one of the miR-16 targets, which enhanced glutamine uptake and the rate of glutaminolysis, which is known to increase in cancer cells. UCA1-induced GLS2 maintained the redox balance and protected cancer cells by reducing excessive ROS production [136]. Oncogenic activity of UCA1 in CRC was the result of its decoy function for miR-204-5p, a critical tumor-suppressive miRNA [137]. UCA1, upregulated in CRC, inhibited miR-204-5p activity, thus promoting the upregulation of miRNA targets CREB1, BCL2, and RAB22A (RAB22A, member RAS oncogene) and regulating cell proliferation and apoptosis [137]. UCA1 upregulation in HCC was associated to cell growth and metastasis; these processes were induced by UCA1 binding to miR-216b and resulted in miR-216b downregulation [138]. Decreased levels of miR-216b led to the derepression of its target FGFR1 (fibroblast growth factor receptor 1) and the activation of ERK pathway [138]. Association between UCA1 and metastatic process was also reported for epithelial ovarian cancer [139]. In fact, UCA1 promoted the expression of MMP14 (matrix metallopeptidase14), a key protein involved in cell invasion, by working as a molecular sponge of miR-485-5p, a miRNA targeting MMP14 [139]. FOXM1 (forkhead box protein M1) is a transcription factor critical for G2/M-phase transition and DNA damage response, and it is also a target of miR-507. UCA1-mediated regulation of FOXM1 was discovered, in melanoma cells, to be based on the ceRNA function of UCA1 for miR-507, resulting in an increased malignant ability of these cells [140].

Finally, a ceRNA role in cancer was also reported for XIST (X-inactivate specific transcript). XIST was the first lncRNA to be functionally characterized, and it is considered the major effector of the X inactivation process during development in female mammals [141]. Its dysregulation was found in several tumors (e.g., breast cancer, glioblastoma, and hepatocellular carcinoma), suggesting that XIST could have a potential diagnostic power in cancer [142–144]. *In vitro* downregulation or upregulation of XIST was associated with altered cell proliferation, metastasis, and apoptosis in several cancer models. Song et al. discovered that XIST overexpression was related to metastasis and poor prognosis of NPC patients [145]. XIST induced the upregulation of E2F3 (E2F transcription factor 3), which is a critical protein for tumor cell proliferation. The authors demonstrated that XIST-promoted activation of E2F3 was caused by the competitive sponge role of XIST for miR-34a-5p (a well-known tumor suppressor miRNA), which targets E2F3 [145]. On the other hand, Chang et al. showed that XIST acts as tumor suppressor and inhibits metastatization and progression in HCC by binding miR-181a and reducing its availability; XIST induces PTEN upregulation, thus decreasing cell proliferation, invasion, and migration [146].

### 2.3. circRNAs as miRNA Sponges

circRNAs are considered new potential players among ceRNAs: they may harbor shared MREs and compete for miRNA binding with mRNAs [69]. Indeed, circRNAs competitively suppress the activity of miRNAs by adsorbing and sequestering them. As miRNAs are strongly involved in nearly all aspects of cellular physiology and perform pivotal roles in initiation and progression of cancer, circRNAs could reasonably be considered as a new class of RNA molecules closely associated with regulation of proliferation, differentiation, and metastatic processes (Table 3).

Zheng et al. reported that circ-TTBK2 (tau tubulin Kinase 2) is significantly upregulated in glioma tissues and cell lines, differently from its linear counterpart [147]. Overexpression of circ-TTBK2 is associated with increased cell proliferation rate, invasion, and decreased apoptosis. Circ-TTBK2 harbors MREs for miR-217, which has a tumor-suppressive role in glioma cells. In fact, circ-TTBK2 and miR-217 interact with each other in an AGO2-dependent manner and upregulation of circ-TTBK2 induced the malignant behavior of glioma cells via downregulation of miR-217. Thus, HNF1*β* (HNF1 homeobox B), a direct target of miR-217, was derepressed and bound to the promoter of Derlin-1 increasing its expression. Finally, Derlin-1 was able to promote cell proliferation, migration, and invasion and inhibit apoptosis of glioma cells by activating PI3K/AKT and ERK pathways. Moreover, restoration of miR-217 expression reversed the circ-TTBK2-induced promotion of cancer progression, suggesting a reciprocal negative feedback between circ-TTBK2 and miR-217 [147].

MiR-145 is a well-known tumor suppressor miRNA in CRC targeting the oncogenes ERK5 (mitogen-activated protein kinase 7) and IRS1 (insulin receptor substrate 1); furthermore, its ability to predict survival of CRC patients was also shown. In a study by Xie et al., it was demonstrated that downregulation of miR-145 in CRC was mechanistically explained by the role of circ_001569 acting as a miRNA sponge to directly inhibit miR-145 action [148]. Circ_001569 was found to be upregulated in CRC tissues and correlated with progression and aggressiveness of the disease. Notably, circ_001569 did not directly affect miR-145 expression, but through a *sponge mechanism* it inhibited its posttranscriptional activity; accordingly, it upregulated its targets E2F5 (E2F transcription factor 5), BAG4 (BCL2-associated athanogene 4), and FMNL2 (formin-like 2), which were responsible for cell proliferation and invasion promotion by circ_001569 [148].

Further work on CRC, investigating the role of cir-ITCH on the biopathology of this cancer, found a potential interaction between cir-ITCH and either miR-7 or miR-20a [149]. Cir-ITCH was downregulated in CRC tissues and its ectopic expression led to decreased cell proliferation. This cellular effect was due to cir-ITCH sponge activity for miR-7 and miR-20a; both can bind the 3′-UTR of ITCH (Itchy E3 Ubiquitin Protein Ligase), which is the linear isoform of cir-ITCH. Cir-ITCH-induced upregulation of ITCH promoted the ubiquitination and degradation of phosphorylated DVL2 (dishevelled segment polarity protein 2) and, accordingly, inhibited the Wnt/*β*-catenin pathway, by repressing the expression of MYC and CCND1 (cyclin D1) [149]. Interestingly, other authors found very similar findings in ESCC: cir-ITCH worked as a miRNA sponge for miR-7, miR-17, and miR-214, increased ITCH expression, and promoted ubiquitin-mediated DVL2 degradation, thus inhibiting canonical Wnt signaling [150].

Besides the cir-ITCH-induced decoy function for miR-7 described above, sponging of miR-7 by CDR1AS was one of the earliest and the most studied ceRNA mechanisms in ncRNA biology, which is also related to cancer. Expression of CDR1AS was found to be elevated in HCC tissues and inversely correlated to miR-7 expression, which was poorly expressed in the same samples [151]. Despite the oncogenic role of miR-7 (previously reported for CRC and ESCC), this miRNA exhibited tumor-suppressive properties in HCC. CDR1AS has sixty-three MREs for miR-7 and strongly suppresses its activity. Knockdown of CDR1AS promoted the expression of miR-7 and suppressed its targets, CCNE1 (cyclin E1) and PIK3CD (phosphatidylinositol-4,5-bisphosphate 3-kinase catalytic subunit delta): this molecular cascade resulted in a reduction of cell proliferation and invasion in HCC [151].

By expression profiling in OSCC, Chen et al. identified the upregulation of a circRNA named circRNA_100290, which was functionally related to abnormal control of cell cycle and cellular proliferation in OSCC cells [152]. circRNA_100290 worked as a miRNA sponge for several members of the miR-29 family, decreasing the quantity of available miR-29s and, accordingly, promoting translation of one of their targets, CDK6 (cyclin-dependent kinase 6), which in turn could induce transition from G1 to S phase in cancer [152].

The first circular transcript identified was Sry circRNA: its encoding gene maps to the sex-determining region of human Y chromosome and was discovered as highly expressed in adult mouse testis [153]. Initially, Sry circRNA was considered an artifact of aberrant RNA splicing and no specific function was attributed to it. The role of Sry circRNA has recently begun to be investigated. Sry circRNA harbors sixteen putative target sites for miR-138 and its function as a miR-138 sponge was demonstrated by Hansen et al. [55]. Currently, no experimental evidence of Sry circRNA-miR-138 axis dysregulation has been reported in cancer; however, as reviewed by Zhao and Shen, miR-138 could target different cancer-related transcripts [154]. For instance, downregulation of miR-138 promoted the malignant progression in cholangiocarcinoma by its target RhoC (ras homolog gene family, member C) [155]. These observations could suggest that the role of competitive endogenous binding between Sry circRNA and miR-138 would be worthy of in-depth analysis in cancer phenotypes.

## 3. Noncoding RNA Network: Future Perspectives for New Therapeutic Approaches

**Figure 1:**
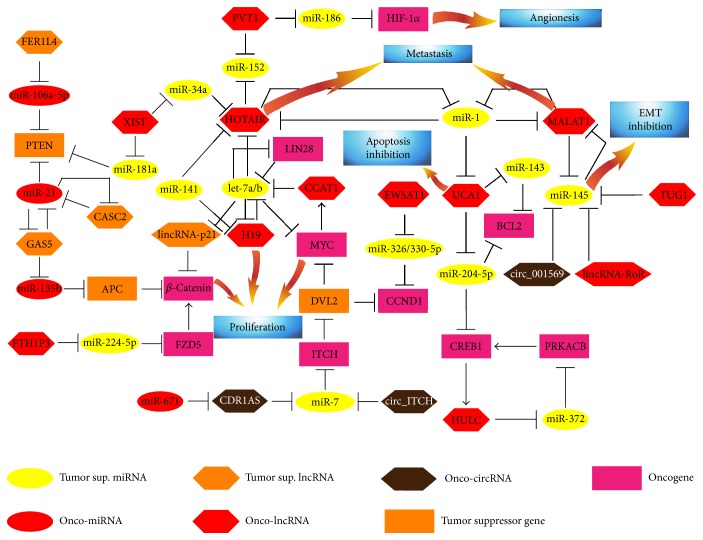
Network of noncoding crosstalking in cancer. Molecular interplay among ncRNAs (i.e., miRNAs, lncRNAs, and circRNAs) in cancer. RNA-RNA interactions were retrieved from papers cited in this review. Lines with arrowheads represent expression activation, those with bars represent expression inhibition.

The existence of a complex RNA-based regulatory signaling, which controls cancer-related pathways, is evident from the experimental evidence collected to date. Such a partially hidden network of RNA-RNA interactions pervades and defines the correct functioning of canonical protein-coding pathways, classically involved in proliferation, differentiation, and invasion in cancer (Figure 1()). The complexity of this *noncoding landscape* is dramatically expanded by the presence of several positive and negative regulatory loops: these make RNA signaling very robust and persistent, though complex and hard to functionally unveil. From a network biology point of view, it is possible to identify some *ncRNA hubs* that are a crossroad among different RNA-based circuits; accordingly, they represent a keystone of network structural integrity. For instance, the tumor suppressor miR-1 could repress and be sponged by the three most potent oncogenic lncRNAs, HOTAIR, UCA1, and MALAT1, which, in turn, could inhibit dozens of miRNAs with tumor-suppressive properties [76, 118, 124]. The signaling passing through let-7a/b appears extremely complex and pronged. Let-7a/b could be considered a crossroad of multiple interplays among cancer-related ncRNAs: let-7a/b and MYC are reciprocally negatively regulated through lncRNA CCAT1 [115], but MYC expression could be indirectly impaired by miR-7, which, in turn, is buffered by different circRNAs [156]. Moreover, let-7a/b could indirectly suppress the *β*-catenin pathway, which in a different way could be activated by lncRNA FTHIP3 [111], but also is regulated by molecular axis miR-21-GAS5-miR-135b [112]. This unexpected crosstalking between ncRNA signaling could shed a light on expression relationships among ncRNAs and mRNAs, which have been frequently reported in cancer literature, but to date have not been satisfactorily explained [157–159]. This *scenario* is made more complex by the tissue-specific expression pattern of all ncRNAs, which could effectively influence the occurrence of specific interactions among ncRNAs. In other words, specific and effective functional interplays among ncRNAs in a particular biological system could occur only if RNA molecules, binding each other, are present at appropriate concentrations. Effectiveness of Ago binding to miRNAs and their targets is dependent on the relative concentration of the miRNA and its target pool [160, 161]. Effective Ago binding occurs when the miRNA : target ratio is close to one but rises dramatically with increasing miRNA : target ratios [162]. Only the most abundant miRNAs show detectable activity, while poorly expressed miRNAs (<100 copies per cell) possess exiguous regulatory properties [163]. However, functional binding between a miRNA and its target can be perturbed by overexpression of other RNAs with multiple shared MREs (e.g., other mRNAs, lncRNAs, and circRNAs) [164]. Such competition among different RNA molecules occurs in a threshold-like manner [165]. Mathematical models predict ceRNA functional effects when miRNA and target levels are near equimolar [166]. However, when the target pool exceeds the threshold set by the buffering miRNA concentration plus the equilibrium dissociation constant (KD) of the miRNA : target interaction, smaller changes in target (i.e., ceRNA) concentration could result in remarkable changes in the concentration of free unrepressed targets [165, 167]. In fact, poorly expressed miRNAs appear to be more susceptible to ceRNA control than more abundant miRNAs. This phenomenon could explain why in *in vitro* experiments a specific miRNA, when ectopically overexpressed, degraded its lncRNA target, but at the same time the enforced upregulation of lncRNA suppressed miRNA activity (e.g., miR-1/MALAT1, miR-21/GAS5) [104, 124]. Taken together, these considerations strongly suggest that miRNA functionality and the switch to ceRNA-promoted repression of miRNAs would be based on the stoichiometric equilibrium among miRNAs and ceRNAs. Based on these observations, physiological ceRNA expression changes could not affect highly expressed miRNAs; however, the relationship between cellular abundance of RNAs and effectiveness of competitive endogenous interactions remains to be fully unveiled in pathological models, in which strong dysregulation of specific ceRNAs could be present [162, 166, 168].

In spite of unclear stoichiometric relationships among ncRNAs in cancer, multiple experimental evidence shows that *in vitro* and *in vivo* modulation of ncRNAs strongly impair aggressive properties of cancer cells. The emerging role of ncRNAs as key regulators of cancer-related signaling makes them very attractive and promising targets for novel, potentially groundbreaking therapeutic approaches. RNA-based therapeutics has several advantages compared to other strategies. RNAs are molecules more *druggable* than proteins, because their targeting is mainly based on nucleic acid complementarity; therefore, an RNA-based drug would be quite easy to design and inexpensive to synthesize (i.e., ASOs, ribozymes, and aptamers) [14]. It is worth stressing that the development of RNA therapeutic strategies has to challenge the redundancy and complexity of the multiple regulatory loops, present in the ncRNA network. It would be quite naive to hypothesize to slow down *in vivo* tumor progression by targeting a single ncRNA molecule: this would be very hard also for protein-based drugs. This axiom should lead researchers to develop multitargeted RNA therapies to improve their impact on oncogenic signaling. In theory, the *β*-catenin pathway, frequently hyperactivated in cancers, could be effectively attenuated by simultaneous silencing of miR-21, miR-135b, and FTH1P3 together with restoring physiologic levels of GAS5, CASC2, and miR-224-5p. Furthermore, simultaneous repression of HOTAIR, MALAT1, and UCA1 with reactivation of miR-1 would result in a pleiotropic favorable effect on different cancer-related processes, such as cancer growth, metastatic behavior, and cell death. Such a synergic approach based on simultaneous administration of miRNA mimics and siRNAs against ncRNAs in *in vitro* and *in vivo* models has already provided encouraging results. Ideally, such therapeutic approaches would be greatly improved by innovative knockout technologies (such as CRISPR/CAS9), which would avoid potential saturation of RISC complexes, typically occurring by using siRNAs or miRNA mimics [169]. The main issue related to ncRNA therapeutics is to develop efficient delivery systems, which should be able to maintain RNA stability in the circulation and guarantee an effective tissue-specific uptake, as well as minimize off-target side effects. Rapid progress in drug delivery technologies has provided promising chemical and nanotechnological resources well adaptable to RNA therapeutics: chemical modifications of antisense molecules (e.g., steroids and cholesterol) [170], adenoviral vectors [171], cationic liposomes [172], and polymer-based nanoparticles [173]. Recently, an exosomal-based miRNA delivery system has been developed. Such a system appears to be very promising because exosomes are less toxic and better tolerated by the organism and naturally protect their molecular cargo in the blood [174].

## 4. Conclusions

A better knowledge on the complex interplay among ncRNAs, together with the development of selective methods for RNA delivery to cancer cells, will provide great benefits for cancer treatment. Needless to say, researchers will have to overcome many technical challenges to develop effective RNA-based anticancer strategies realistically applicable to patients. Before ncRNA targeting is pervasively applied in clinical settings, it will be indispensable to organize large collaborative efforts between research institutes and industry to fully realize the clinical potential of this very promising approach.

## Figures and Tables

**Table 1:** miRNAs inducing degradation of lncRNAs.

miRNA	lncRNA/circRNA target	Tumor	miRNA role	PMID
let-7b	lincRNA-p21	Cervical carcinoma	Tumor suppressor	22841487
let-7b, let-7i	HOTAIR	Cervical carcinoma	Tumor suppressor	24326307
miR-1	UCA1	Bladder cancer	Tumor suppressor	25015192
miR-9	MALAT1	Hodgkin lymphoma, glioblastoma	Tumor suppressor	23985560
miR-21	CASC2	Renal cell carcinoma	Oncogene	27222255
miR-21	CASC2	Glioblastoma	Oncogene	25446261
miR-21	GAS5	Breast cancer	Oncogene	23933812
miR-34a	HOTAIR	Prostate cancer	Tumor suppressor	23936419
miR-101	MALAT1	Esophageal squamous cell carcinoma	Tumor suppressor	25538231
miR-125b	HOTTIP	Hepatocellular carcinoma	Tumor suppressor	25424744
miR-125b	MALAT1	Bladder cancer	Tumor suppressor	24396870
miR-141	H19	Gastric cancer	Tumor suppressor	26160158
miR-141	HOTAIR	Renal carcinoma	Tumor suppressor	24616104
miR-217	MALAT1	Esophageal squamous cell carcinoma	Tumor suppressor	25538231
miR-671	CDR1AS	Glioblastoma	Oncogene	26683098

This table reports for each miRNA: (1) its lncRNAs/circRNA target; (2) tumor where such interaction was reported; (3) its function in cancer (oncogene or tumor suppressor); and (4) bibliographic reference reported as Pubmed ID (PMID).

**Table 2:** lncRNAs acting as decoy of miRNAs.

lncRNA	miRNA target	Tumor	lncRNA role	PMID
CCAT1	let-7	Hepatocellular carcinoma	Oncogene	25884472
EWSAT1	miR-326/−330-5p cluster	Nasopharyngeal carcinoma	Oncogene	27816050
FER1L4	miR-106a-5p	Gastric cancer	Tumor suppressor	26306906
FTH1P3	miR-224-5p	Squamous cell carcinoma	Oncogene	28093311
FTX	miR-374a	Hepatocellular carcinoma	Tumor suppressor	27065331
GAS5	miR-135b	Non-small cell lung cancer	Tumor suppressor	28117028
H19	let-7a, let-7b	Breast cancer	Oncogene	28102845
HOST2	let-7b	Epithelial ovarian cancer	Oncogene	25292198
HOTAIR	miR-1	Hepatocellular carcinoma	Oncogene	27895772
HOTAIR	miR-152	Gastric cancer	Oncogene	26187665
HULC	miR-372	Liver cancer	Oncogene	20423907
lincRNA-RoR	miR-145	Breast cancer	Oncogene	25253741
lincRNA-RoR	miR-145	Endometrial cancer	Oncogene	24589415
LOC100129148	miR-539-5p	Nasopharyngeal carcinoma	Oncogene	28328537
MALAT1	miR-1	Breast cancer	Oncogene	26676637
MALAT1	miR-145	Cervical cancer	Oncogene	26311052
NEAT1	miR-449-5p	Glioma	Oncogene	26242266
PVT1	miR-152	Gastric cancer	Oncogene	28258379
PVT1	miR-186	Gastric cancer	Oncogene	28122299
RMRP	miR-206	Gastric cancer	Oncogene	27192121
SPRY4-IT1	miR-101-3p	Bladder cancer	Oncogene	27998761
TUG1	miR-145	Bladder cancer	Oncogene	26318860
TUG1	miR-299	Glioblastoma	Oncogene	27345398
TUG1	miR-300	Gallbladder carcinoma	Oncogene	28178615
TUG1	miR-9-5p	Osteosarcoma	Oncogene	27658774
TUSC7	miR-10a	Hepatocellular carcinoma	Tumor suppressor	27002617
TUSC7	miR-211	Colon cancer	Tumor suppressor	23558749
TUSC7	miR-23b, miR-320d	Gastric cancer	Tumor suppressor	25765901
UCA1	miR-143	Breast cancer	Oncogene	26439035
UCA1	miR-16	Bladder cancer	Oncogene	26373319
UCA1	miR-204-5p	Colorectal cancer	Oncogene	27046651
UCA1	miR-216b	Hepatocellular carcinoma	Oncogene	25760077
UCA1	miR-485-5p	Epithelial ovarian cancer	Oncogene	26867765
UCA1	miR-507	Melanoma	Oncogene	27389544
XIST	miR-139-5p	Hepatocellular carcinoma	Oncogene	28231734
XIST	miR-181a	Hepatocellular carcinoma	Tumor suppressor	28388883
XIST	miR-34a-5p	Nasopharyngeal carcinoma	Oncogene	27461945
XIST	miR-92b	Hepatocellular carcinoma	Tumor suppressor	27100897

This table reports for each lncRNA: (1) miRNA sponged; (2) tumor where such interaction was reported; (3) its function in cancer (oncogene or tumor suppressor); and (4) bibliographic reference reported as Pubmed ID (PMID).

**Table 3:** circRNAs acting as miRNA sponges.

circRNA	miRNA target	tumor	circRNA role	PMID
circRNA_0005075	miR-23b-5p, miR-93-3p, miR-581, miR-23a-5p	Hepatocellular carcinoma	Oncogene	27258521
circRNA_001569	miR-145	Colorectal cancer	Oncogene	27058418
circRNA_100290	miR-29 family	Oral cancer	Oncogene	28368401
Cdr1as	miR-7	Hepatocellular carcinoma	Oncogene	27391479
cir-ITCH	miR-7, miR-20a	Colorectal cancer	Tumor suppressor	26110611
cir-ITCH	miR-7, miR-17, miR-214	Esophageal squamous cell carcinoma	Tumor suppressor	25749389
ciR-SRY	miR-138	Cholangiocarcinoma	Oncogene	27671698, 23446431
cir-TTBK2	miR-217	Glioma	Oncogene	28219405

This table reports for each circRNA: (1) miRNAs sponged; (2) tumor where such interaction was reported; (3) its function in cancer (oncogene or tumor suppressor); and (4) bibliographic reference reported as Pubmed ID (PMID).
